# Efficacy of interferon alpha-2b with or without ribavirin in thalassemia major patients with chronic hepatitis C virus infection: A randomized, double blind, controlled, parallel group trial

**Published:** 2010

**Authors:** Hamid Kalantari, Neda Rad

**Affiliations:** a Associate Professor, Department of Gastroenterology, School of Medicine, Isfahan University of Medical Sciences, Isfahan, Iran; b Resident of Internal Medicine, School of Medicine, Isfahan University of Medical Sciences, Isfahan, Iran

**Keywords:** Hepacivirus, Interferons, Ribavirin, Thalassemia

## Abstract

**BACKGROUND:**

The aim of this study was to evaluate the effectiveness of monotherapy with interferon alpha-2b and combination therapy with interferon alpha-2b plus ribavirin on chronic hepatitis C infection in thalassaemic patients.

**METHODS:**

In parallel group randomized, double blind, controlled trial, 32 thalassaemic patients with chronic hepatitis C infection completed the study. In a random fashion, one group was treated with three million units of interferon alpha-2b three times a week plus ribavirin (800-1200 mg daily). The second group received interferon alpha-2b alone. Treatment duration was 24-48 weeks. Primary efficacy variables were HCV RNA after treatment and sustained viral response (SVR) six months after treatment.

**RESULTS:**

The mean age of patients was 22 ± 7.4 years; 19 (59.4%) were male and 13 (40.6) were female. At the end of treatment, no statistically significant differences were found between the groups in HCV RNA and AST. The proportion of patients with SVR six months after treatment was significantly greater in the monotherapy group (90.9%) than in the combination therapy group (44.4%; p = 0.049). A significant difference in mean of ALT was also obtained at the end of treatment between monotherapy and combination therapy groups (30.4 ± 19.2 and 60.1 ± 48.9, respectively; p = 0.02). Response rates were not associated with genotype and severity of hepatitis C infection in both groups.

**CONCLUSIONS:**

These results suggest that monotherapy may be considered as the first-line therapy in patients with thalassemia.

About 75 percent of patients with acute hepatitis C ultimately develop chronic infection. Hepatitis C is the cause of about half of cases of primary liver cancer in the developed world.^1^,^2^ Hepatitis C virus infection is common in patients receiving long-term blood transfusion therapy.^3^ Patients with β-thalassemia major receive chronic blood transfusions and have an increased prevalence of chronic hepatitis C virus infection.^4^ The prevalence of chronic hepatitis C infection ranges from 25 to 75% in thalassemic patients.^5^ Treatment strategies have evolved from monotherapy with interferon alpha-2b to combination therapy with ribavirin.^6^

Several studies evaluated the effects of interferon alpha and ribavirin on chronic hepatitis infection in thalassemic patients. Telfer et al have conducted a pilot study of combination antiviral therapy for 11 patients who failed to respond, or relapsed after an initial response to single-agent interferon-alpha. Patients were treated for six months with interferon alpha-2b, with ribavirin. They showed that five patients (45.5%) had a sustained virological response (SVR) with loss of serum HCV RNA for > 6 months after finishing therapy. Transfusion requirements were significantly increased during the treatment phase. They suggest that combination therapy is valuable in clearing HCV infection, and may provide effective second-line therapy for thalassemic patients who have failed to respond to interferon-alpha monotherapy.^7^ Inati et al in 2005 evaluated 20 patients and demonstrated that SVR occurred in 30% and 62.5% of patients in the monotherapy and combination groups, respectively. Age < 18 years were associated with improved SVR.^8^ Harmatz et al evaluated 16 thalassemic patients with chronic hepatitis infection at the year of 2008. One of four patients (25%) with genotype 2 or 3 and six of 12 patients (50%) with genotype 1 demonstrated sustained viral response; and suggest that combination therapy is safe if transfusion requirement, iron toxicities and neutropenia are monitored.^9^ Ancel et al 2009, showed that SVR was achieved in 45.5% of patients (5/11) after combination therapy.^10^

One case report study in 2005, reported SVR for nearly 24 months after combination therapy.^11^ Some studies published that most of thalassemic patients had SVR six months after monotherapy.^12^,^13^ Another studies reported SVR six months after combination therapy.^14^,^15^

Although investigators have reported increase in SVR after combination antiviral therapy versus monotherapy, there are not enough data from parallel-group randomized controlled trials assessing its efficacy. The aim of this study was to test the hypothesis that combined treatment with interferon alpha-2b, plus ribavirin would be differ from interferon alpha-2b alone for chronic hepatitis infection in thalassemic patients, in a parallel-group randomized controlled trial.

## Methods

This study was a parallel-group randomized controlled trial, from August 2007, to August 2009, conducted in the Center of Research for Hepatitis in Sayed-al-Shohada Hospital in Isfahan University of Medical Sciences. Ethical approval was obtained from the local research ethics committee in school of medicine before recruitment. The study was done in accordance with published guidelines^16^ and is reported in accordance with CONSORT guidelines.

Patients were eligible for enrolment if they had a clinical diagnosis of thalassemia, chronic hepatitis C or increment of ALT and AST in serum, but excluded if they were pregnant, or had immunocompromised patients (such as patients with human immunodeficiency virus infection), renal failure, serological evidence of active hepatitis B virus infection, clinical evidence of hepatic decompensation, neutropenia < 1 × 10^9^ /l and thrombocytopenia < 100 × 10^9^ /l. The main objective was to explore if addition of ribavirin to interferon alpha-2b to treatment of chronic hepatitis C virus infection in thalassemic patients would improve SVR compared with interferon alpha-2b treatment alone. Thirty seven patients were defined as eligible patients. Written informed consent was recorded from subjects who agreed to participate. One patient refused consent. Thirty six patients were randomly assigned to two groups using a computer-generated number list. First group (n = 18) received interferon alpha-2b (three million units three times a week) plus ribavirin (patients with genotype 3 received 800 mg daily, patients with genotype1 and nontypable received 1000 mg daily if their body weight was < 75 kg and 1200 mg daily for patients who were > 75 kg). And second group (n = 18) received interferon alpha-2b (three million units three times a week). A liver biopsy was performed before the start of treatment. And were graded and staged according to the Knodell scoring (HAI).^17^ Treatment duration was 24 weeks for patients with genotype 3 and 48 weeks for genotype 1 and nontypable patients.

The primary end-point was HCV RNA, which was measured at the end of treatment and 6 months after finishing therapy. AST and ALT measured as secondary end-points at the end of treatment. Patients visited the clinic once a week at the first month and then once a month to assess clinical status. Patients who had negative HCV RNA after treatment were followed-up for at least 6 months after completion of their course of treatment to assess SVR result. Patients, health-care professionals and project analyst were unaware of the treatment assigned. To achieve this, patients in mono-therapy group received placebo daily instead of ribavirin.

Due to the sample size, non-parametric data analysis was performed. Data are presented as means ± 1SD or median (IQR) as appropriate. All analyses were done with the use of SPSS software (version 17). Outcomes were compared between the groups using Chi square test and Fisher Exact test. Mann-Whitney U test was used to compare responses between the treatment strategies. P value of less than 0.05 was regarded as statistically significant.

## Results

**Figure 1:**
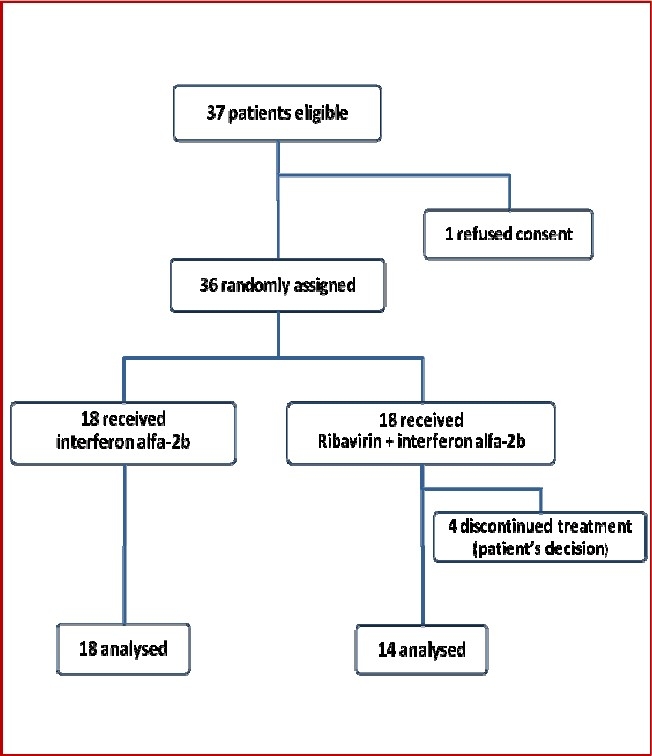
Trial profile

Between August 2007, and August 2009, 36 patients were randomly assigned: 18 were assigned to receive combination therapy and 18 to receive interferon alpha-2b alone. Figure 1 shows the trial profile. All patients in the monotherapy group were included in the analysis. But among the patients in the combination therapy group, 4/18 did not receive treatment (due to patient’s decision) and 14/18 patients were included in the analysis. Subjects who did not complete the study did not differ in terms of baseline characteristics from those who completed the study (data not shown).

The mean age of patients was 22 ± 7.4 years (range: 8-45), 19 (59.4%) were male and 13 (40.6) were female. Overall in both groups 16 (50%) patients had genotype 1, 14 (43.7%) had genotype 3 and 2 (6.3%) were nontypable. Five patients (15.6%) refused liver biopsy, the mean of Knodell score for patients in the combination therapy group was 15 ± 5.1 and for patients in the monotherapy therapy group was 12.44 ± 5. Table 1 shows the demographic and baseline clinical characteristics of the study population. Characteristics of patients were well balanced in the two groups. There were no significant differences between the two treatment groups for any of these characteristics.

**Table 1:** Demographic and clinical characteristics in 32 thalassemic patients

	IR (n = 14)	I (n = 18)	P value*
Age (years)	21.5 (18-23.25)	20.5 (18-24.5)	0.9
Sex:			
Female	7 (50)	12 (66.7)	0.34
Male	7 (50)	6 (33.3)	
Genotype:			
1	8 (57.1)	8 (44.5)	0.41
3	6 (42.9)	8 (44.5)	
Nontypable	0	2 (11)	
Knodell score	15 ± 5.1	12.44 ± 5	0.2

Data are median (IQR), number (%) and mean ± 1SD.

IR = Interferon + Ribavirin; I = Interferon

* Mann-Whitney test and Chi square test were used for p values.

HCV RNA for 20 (62.5%) patients was negative and for 12 patients (37.5%) was positive at the end of treatment. The results for the primary and secondary outcomes are shown in table 2. There was no significant difference between study groups in the percentage of patients who had negative HCV RNA after treatment (p = 0.8). Also analysis of the secondary outcomes showed that there was no significant difference in the mean of AST between groups. But it was found that mean of ALT for patients who received interferon plus ribavirin was significantly higher than the mean of ALT for patients with monotherapy (p = 0.03). Other primary end-point was SVR that calculated six months after treatment for patients who had negative HCV RNA after treatment. Fourteen out of 20 patients (70%) had SVR and 6 out of 20 patients (30%) didn’t have SVR. Ten out of 11 (90.9%) patients in interferon plus ribavirin group and four out of nine (44.4%) in interferon group had SVR after six months (Table 3). To assess the difference of SVR between groups, Fisher Exact test was used indicating significant difference between treatment groups (p = 0.049). Additional subgroup analyses were done for genotype of HCV and severity of HCV and are shown in table 3. There was no association between subgroups for SVR (p > 0.05).

**Table 2:** Clinical data after treatment in 32 thalassemic patients

	IR (n = 14)	I (n = 18)	P value*
AST (IU/L)	59.7 ± 58.5	38.9 ± 24.7	0.26
ALT (IU/L)	60.1 ± 48.9	30.4 ± 19.2	0.03
HCV RNA:			
Negative	9 (64.3)	11 (61.1)	0.8
Positive	5 (35.7)	7 (38.9)	

Data are mean ° 1SD and number (%).

IR = Interferon + Ribavirin; I = Interferon

* P value calculated with Mann-Whitney test.

**Table 3:** Frequency of patients who had SVR and its association with genotype of chronic hepatitis C in 20 thalassemic patients with negative HCV RNA after treatment

	n	IR (n =9)	I (n = 11)	P value*
SVR	20	4/9	10/11	0.049
Genotype:				
1	10	2/5	4/5	NS
3	9	2/4	5/5	
Nontypable	1	0	1/1	

IR = Interferon + Ribavirin; I = Interferon

* Fisher Exact test was used for p value

NS: not significant

**Figure 2:**
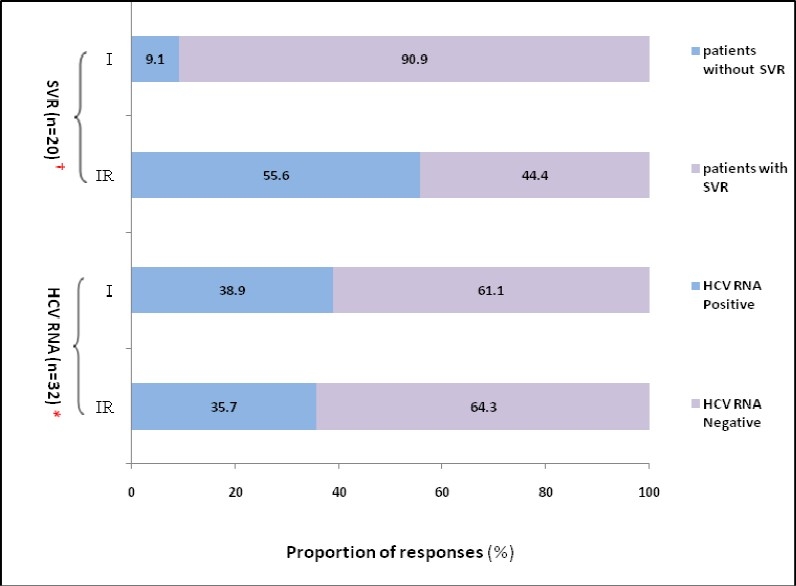
Outcome for each group during the study (proportion of responses to HCVRNA and SVR after treatment) IR = Received Interferon alpha-2b plus Ribavirin I = Received Interferon alpha-2b alone † n = 20: Number of patients who had negative HCVRNA at the end of treatment and six month follow up after treatment * n = 32: Number of thalassemic patients with chronic hepatitis C infection who completed the study

Figure 2 shows the outcome for each group during the study. In HCV RNA columns proportion of responses for 32 patients are shown. The result of treatments was similar in two groups of trial at the end of treatment and there was no significant difference between two regimes. In SVR columns proportion of responses for 20 patients who had negative HCV RNA are shown. And it seems that the number of patients who had SVR 10/11 (90.9%) in monotherapy group is more than the number of patients who had SVR 4/9 (44.4%) in combination therapy group. Indeed the relapse rate is lower in patients treated with monotherapy compared with combination therapy. There was statistically significant difference between two regimes.

## Discussion

The present study indicates a significant superiority of the interferon alpha-2b treatment alone compared with interferon alpha-2b plus ribavirin treatment for SVR in thalassemic patients with chronic hepatitis C virus. No difference was observed in the frequency of HCV RNA responses between treatment groups. But a significant difference was identified in the frequency of SVR six months after treatment between groups. Also ALT for patients who received interferon plus ribavirin was higher than the patients with monotherapy.

It was shown that 61.1% of patients had negative HCV RNA in monotherapy group, which is in line with those of two small controlled but non-randomized studies that show HCV RNA for monotherapy treatment was 80% and 36-56%.^12^,^13^ Also the present findings showed 44.4% for SVR in combination therapy group that are similar to those studies that reported 45.5%, 50%, 45.5%, 50-55% and 72.2%.^7^,^9^,^10^,^14^,^15^ Although unlike these studies, the present results were obtained from parallel-group randomized trial.

Inati et al^8^ in a double-blind parallel-group randomized controlled trial showed that rate of SVR in combination therapy group was more than monotherapy group (62.5% vs. 30%, p = 0.19); and suggest that the combination therapy is effective, probably safe, and should arguably be considered as the first-line therapy in patients with thalassemia. But it was found that SVR in monotherapy group was more than combination therapy group (90.9% vs. 44.4% p = 0.049). Therefore the present results are not in agreement with Inati et al results that the combination therapy can be used as the first-line therapy in thalassemic patients. Telfer et al^7^ and Hamidah et al^11^ study suggest that the combination therapy may provide effective second-line therapy for thalassaemic patients who have failed to respond to interferon-alpha monotherapy. However, there are other studies that advise the combination therapy as the first-line therapy.^9^,^10^

Butensky et al^14^ demonstrated that the rate of SVR in patients with genotypes 2 and 3 is higher than the other genotypes. Unlike Telfer et al,^7^ in the present study no association was found between SVR with patients genotypes.

A weakness of the present study is the small number of patients enrolled primarily as a result of the declining number of patients with thalassemia and HCV who are eligible. Another shortcoming of the present study is the lack of information on transfusion requirements.

These results cannot directly be advised to patients; and more trials addressing this issue need to be undertaken.

## Conclusions

In conclusion the present results show that monotherapy is more effective than combination therapy in thalassemic patients with chronic hepatitis C virus. And monotherapy may be considered as the first-line therapy in patients with thalassemia. Then combination therapy may provide effective second-line therapy for patients who have failed to respond to interferon-alpha monotherapy.
